# Neuroprotective Effects of Inhibiting Fyn *S*-Nitrosylation on Cerebral Ischemia/Reperfusion-Induced Damage to CA1 Hippocampal Neurons

**DOI:** 10.3390/ijms17071100

**Published:** 2016-07-12

**Authors:** Lingyun Hao, Xuewen Wei, Peng Guo, Guangyi Zhang, Suhua Qi

**Affiliations:** 1 Research Center for Biochemistry and Molecular Biology, and Jiangsu Key Laboratory of Brain Disease Bioinformation, Xuzhou Medical University, Xuzhou 221002, China; hlyxuzhou@163.com (L.H.); xuewenwei@126.com (X.W.); gpxuzhou8912@163.com (P.G.); 2 Jiangsu Key Laboratory of Anesthesia and Analgesia Application Technology, Xuzhou 221002, China; 3 Department of Laboratory Medicine, Affiliated Municipal Hospital of Xuzhou Medical University, Xuzhou 221002, China

**Keywords:** cerebral ischemia and reperfusion, Fyn, *S*-nitrosylation, phosphorylation, neuroprotection

## Abstract

Nitric oxide (NO) can regulate signaling pathways via *S*-nitrosylation. Fyn can be post-translationally modified in many biological processes. In the present study, using a rat four-vessel-occlusion ischemic model, we aimed to assess whether Fyn could be *S*-nitrosylated and to evaluate the effects of Fyn *S*-nitrosylation on brain damage. In vitro, Fyn could be *S*-nitrosylated by *S*-nitrosoglutathione (GSNO, an exogenous NO donor), and in vivo, endogenous NO synthesized by NO synthases (NOS) could enhance Fyn *S*-nitrosylation. Application of GSNO, 7-nitroindazole (7-NI, an inhibitor of neuronal NOS) and hydrogen maleate (MK-801, the *N*-methyl-d-aspartate receptor (NMDAR) antagonist) could decrease the *S*-nitrosylation and phosphorylation of Fyn induced by cerebral ischemia/reperfusion (I/R). Cresyl violet staining validated that these compounds exerted neuroprotective effects against the cerebral I/R-induced damage to hippocampal CA1 neurons. Taken together, in this study, we demonstrated that Fyn can be *S*-nitrosylated both in vitro and in vivo and that inhibiting *S*-nitrosylation can exert neuroprotective effects against cerebral I/R injury, potentially via NMDAR-mediated mechanisms. These findings may lead to a new field of inquiry to investigate the underlying pathogenesis of stroke and the development of novel treatment strategies.

## 1. Introduction

Fyn (EC = 2.7.10.2) is a member of the Src family of non-receptor protein tyrosine kinases (SrcPTKs), and it has been reported to possess three transcript isoforms: isoform 1 (Fyn (B)), isoform 2 (Fyn (T)) and isoform 3. Isoform 2 tends to be expressed in T cells. The expression of Fyn (B) is the most ubiquitous and is highly expressed in the brain [1,2,3,4]. As a signal integration hub, Fyn plays important roles in neuronal functions by regulating signaling pathways, especially in the development of severe brain pathologies such as stroke and Alzheimer’s disease [5]. Fyn (B) is a 59-kDa protein that comprises 537 amino acids, including nine cysteine residues (at positions 3, 6, 239, 240, 246, 404, 487, 491, and 502). The protein has a similar structure to Src, which can be *S*-nitrosylated via the cysteine residue at position 498 [6]. In addition, our previous study has shown that cerebral ischemia/reperfusion (I/R) can induce the *S*-nitrosylation of Src in hippocampal CA1 neurons, a process mediated by neuronal nitric oxide synthase (nNOS) [7]. Whether Fyn can be *S*-nitrosylated during cerebral I/R is unknown.

Global ischemia and reperfusion can facilitate a massive increase in glutamate release, which may activate glutamate receptors, including the *N*-methyl-d-aspartate receptor (NMDAR). In ischemic neurons, glutamate increases intracellular Ca^2+^ concentrations through activation of NMDAR, leading to a persistent increase in the activity of nNOS [8,9]. nNOS activation can lead to generation of endogenous NO, which intermediately activates guanylate cyclase, resulting in an increase in cyclic guanosine monophosphate (cGMP) and activation of downstream signaling pathways [10]. NO can also regulate cell signaling pathways via post-translational modification of proteins. This modification is referred to as *S*-nitrosylation. The addition of a NO group to a cysteine residue results in the formation of an *S*-nitrosoprotein, which can play critical roles in various physiological and pathological functions [11,12,13]. Increasing evidence suggests that dysregulation of *S*-nitrosylation may be involved in the pathophysiological processes of numerous disorders, particularly degenerative diseases affecting the central nervous system [14,15]. Therefore, identifying potential targets of *S*-nitrosylation and better understanding their associated regulatory mechanisms may contribute to the development of therapeutic interventions for these neurological diseases. The regulation of NMDA receptors occurs largely via the phosphorylation and dephosphorylation of the NMDA Receptor 2 (GluN2) subunits. Fyn can phosphorylate NMDA Receptor 2B (GluN2B) at Y1252, Y1336, and Y1472, thereby altering the trafficking to the plasma membrane and degradation of NMDAR, and it acts as a point of convergence in the regulation of NMDARs via many signaling pathways [16]. Considering that Fyn shares a similar structure to Src, we hypothesized that the formation of *S*-nitrosylated Fyn was possible.

In the present study, we proposed to identify whether Fyn can be *S*-nitrosylated and to investigate the effects of the *S*-nitrosylation of Fyn on brain damage. We further elucidate the underlying molecular mechanisms of Fyn regulation. First, we determined whether Fyn underwent *S*-nitrosylation following treatment with *S*-nitrosoglutathione (GSNO) in Fyn-overexpressing HEK293 cells. We then measured the levels of Fyn *S*-nitrosylation induced by cerebral I/R at various time points after reperfusion. We also observed the effects of various compounds, including GSNO, 7-nitroindazole (7-NI, an inhibitor of neuronal nitric oxide synthase (nNOS)) and hydrogen maleate (MK-801, an NMDAR antagonist) on Fyn *S*-nitrosylation and activation. These compounds were further evaluated for their effects on the damage to rat hippocampal CA1 neurons after cerebral I/R.

## 2. Results

### 2.1. Fyn Was S-Nitrosylated by *S*-Nitrosoglutathione (GSNO) in HEK293 Cells

**Figure 1:**
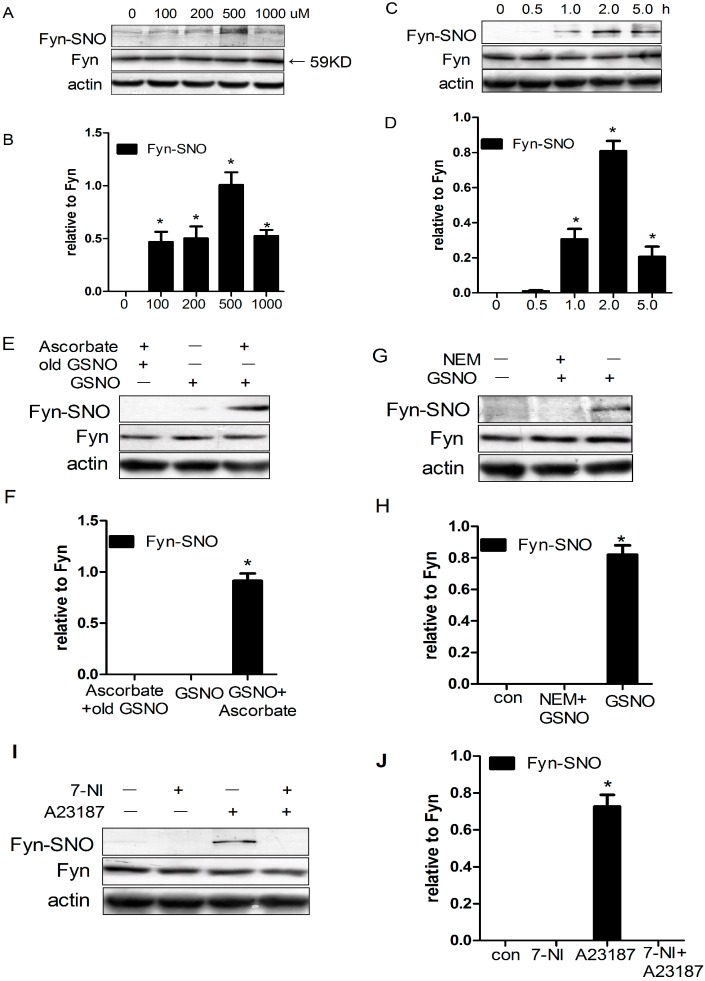
Fyn (*M*_W_ = 59 KD) was *S*-nitrosylated by *S*-nitrosoglutathione (GSNO) in HEK293 cells. (**A**) HEK293 cells overexpressing Fyn were treated with GSNO at 0, 100, 200, 500, and 1000 μM for 2 h and assessed for Fyn *S*-nitrosylation; (**B**) Data are expressed as the means ± S.D. (*n* = 4). * p < 0.05 compared with the basal level; (**C**) HEK293 cells overexpressing Fyn were treated with 500 μM GSNO for 0, 0.5, 1, 2, and 5 h and evaluated for time-dependent patterns of Fyn *S*-nitrosylation by GSNO; (**D**) Data are presented as the means ± S.D. (*n* = 4). * p < 0.05 compared with the basal level; (**E**) After serum starvation for 2 h, HEK293 cells overexpressing Fyn were treated with either 500 μM inactive GSNO or 500 μM GSNO, as indicated, for 2 h. Fyn *S*-nitrosylation was assessed in the absence or presence of ascorbate, as indicated; (**F**) Data are expressed as the means ± S.D. (*n* = 4). * p < 0.05 compared with the ascorbate plus inactive GSNO group; (**G**) HEK293 cells overexpressing Fyn were treated with or without *N*-ethylmaleimide (NEM) in the absence of serum for 2 h, followed by administration of 500 μM GSNO for 2 h; (**H**) Data are expressed as the means ± S.D. (*n* = 4). * p < 0.05 compared with the control group; (**I**) HEK293 cells co-overexpressing Fyn and nNOS were deprived of serum for 2 h, then treated with the presence or absence of 7-nitroindazole (7-NI), and then treated with A23187; (**J**) Data are expressed as the means ± S.D. (*n* = 4). * p < 0.05 vs. the control group.

To verify whether Fyn can be *S*-nitrosylated in vitro, HEK293 cells were transfected with wild-type Fyn plasmid and stimulated with various concentrations of GSNO (0, 100, 200, 500, or 1000 μM) for 2 h. Cells were then lysed, and the levels of *S*-nitrosylated Fyn were detected using immunoprecipitation (IP) with anti-Fyn antibody, followed by immunoblotting (IB) with anti-SNO-Cys antibody and western blotting in the presence of ascorbate. Upon treatment with GSNO at 100–500 μM, Fyn was *S*-nitrosylated in a concentration-dependent manner, with the most potent effect in HEK293 cells produced by treatment at 500 μM GSNO (Figure 1A,B). However, upon treatment with 1000 μM GSNO, the Fyn *S*-nitrosylation reverted back. This suggested that physiologically low concentrations of GSNO (100–500 μM) resulted in dose-dependent increases in the *S*-nitrosylation of Fyn. However, high concentrations of “NO donors” (at nitrosative stress-associated concentrations) decreased the Fyn *S*-nitrosylation.

Next, we evaluated the time-dependent effects of GSNO treatments on Fyn *S*-nitrosylation in transfected HEK293 cells by administering 500 μM GSNO for various durations. Treatment with GSNO increased Fyn *S*-nitrosylation in a time-dependent manner, with the maximum level occurring at 2 h of continuous administration (Figure 1C,D). In contrast, treatment of transfected cells with inactivated GSNO (which had been exposed to light for at least 24 h) failed to induce Fyn *S*-nitrosylation. Treatment with GSNO without ascorbate, which was treated as a negative control, failed to induce Fyn *S*-nitrosylation (Figure 1E,F). Additionally, 30 min of pretreatment with *N*-ethylmaleimide (NEM), a sulfhydryl alkylating agent, to block the free thiol groups of Fyn cysteine residues reversed GSNO-induced Fyn *S*-nitrosylation (Figure 1G,H).

We additionally co-expressed wild-type Fyn with nNOS (a calcium-calmodulin-regulated, A23187-activated enzyme) in HEK293 cells and monitored Fyn *S*-nitrosylation upon treatment with either 7-NI (a nNOS inhibitor) or the calcium ionophore A23187. We found that A23187 significantly enhanced Fyn *S*-nitrosylation, while 7-NI prevented this type of posttranslational modification (Figure 1I,J). The present results suggest that Fyn could be *S*-nitrosylated by endogenous NO produced by nNOS in HEK293 cells.

### 2.2. S-Nitrosylation and Phosphorylation of Fyn Are Mediated by Nitric Oxide Synthase (nNOS), with Potential Involvement of N-Methyl-d-aspartate Receptors (NMDARs) in Vivo

**Figure 2:**
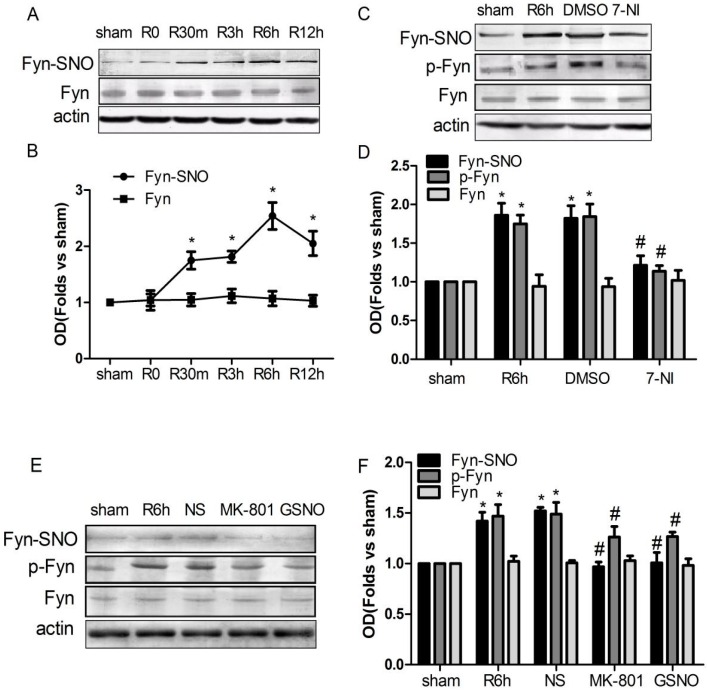
The *N*-methyl-d-aspartate receptor (NMDAR)/ nitric oxide synthase (nNOS) signaling module may have been involved in the S-nitrosylation and phosphorylation of Fyn. (**A**,**B**) Time course of *S*-nitrosylated Fyn levels in hippocampal CA1 in rats after sham treatment or 15 min of ischemia at different time points during reperfusion. * *p <* 0.05 vs. sham; (**C**,**E**) Effects of 7-NI, MK-801 and GSNO on Fyn *S*-nitrosylation and phosphorylation at 6 h of reperfusion after ischemia. The levels of phosphorylated and total Fyn were determined by immunoblotting using antibodies specific for phosphorylated and total Fyn, respectively; (**D**,**F**) Relative levels of *S*-nitrosylated, phosphorylated and total Fyn compared with the sham group. Data are presented as the means ± S.D. (*n* = 4). * p < 0.05 vs. sham, *# p* < 0.05 vs. the corresponding solvent-treated control group.

Chen et al. [17] reported that cerebral ischemia for 15 min significantly increased the phosphorylation of Fyn at 6 h after reperfusion. We therefore examined the effect of time following I/R on Fyn *S*-nitrosylation in hippocampal CA1 neurons. We found that reperfusion following cerebral ischemia resulted in a time-dependent increase in Fyn *S*-nitrosylation as early as 15 min after reperfusion (without affecting total Fyn expression), with a maximum increase occurring at 6 h following reperfusion (Figure 2A,B). We also treated rats with 7-NI, a nNOS inhibitor, for 20 min prior to 6 h of reperfusion after 15 min of ischemia. As shown in Figure 2C,D, I/R-induced Fyn *S*-nitrosylation and phosphorylation were inhibited by treatment with 7-NI. These data suggest that the induction of Fyn *S*-nitrosylation and phosphorylation was mediated by endogenous NO, which was produced by nNOS during cerebral I/R.

As shown in Figure 2E,F, pretreatment with the NMDAR antagonist MK-801 (a highly potent, selective and non-competitive open channel blocker) partially attenuated the I/R-induced Fyn phosphorylation and *S*-nitrosylation without affecting total Fyn protein levels. These results indicate the likely involvement of NMDARs in this process.

We further investigated the effects of exogenous NO on Fyn activation and *S*-nitrosylation induced by cerebral I/R by treating rats with the exogenous NO donor GSNO. Administration of GSNO significantly decreased Fyn *S*-nitrosylation and phosphorylation but had no effect on total Fyn protein levels in vivo (Figure 2E,F).

### 2.3. Treatment with Drugs that Attenuate Cerebral Ischemia/Reperfusion (I/R)-Induced Fyn S-Nitrosylation Results in Neuroprotection

**Figure 3:**
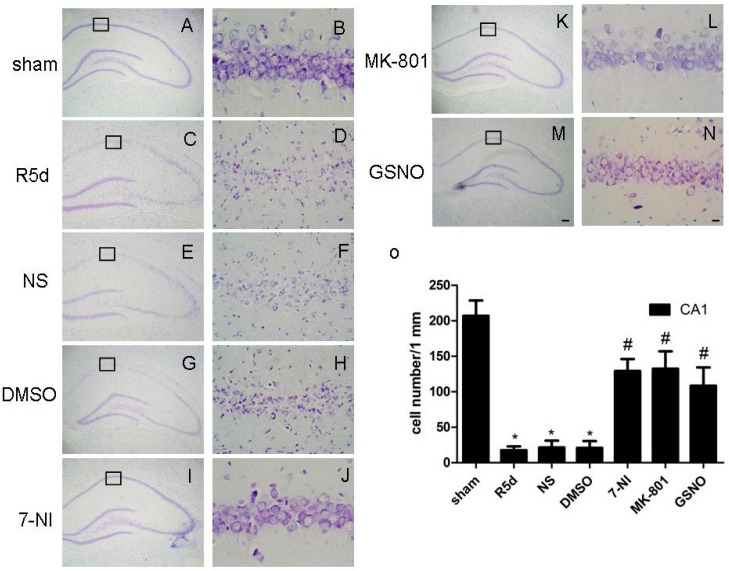
GSNO, MK-801, and 7-NI have neuroprotective roles against injury induced by cerebral ischemia/reperfusion (I/R). Cresyl violet-stained sections from the hippocampi of: sham-operated rats (**A**,**B**); rats subjected to ischemia and five days of reperfusion (**C**,**D**); vehicle-treated rats (**E**–**H**); and rats treated with 7-NI (**I**,**J**), GSNO (**K**,**L**), or MK-801 (**M**,**N**); (**O**) Quantitative analysis of the protective effects of 7-NI, MK-801 and GSNO on the damage to hippocampal neurons after cerebral I/R. Data were obtained from four independent animals, and typical experiment results are shown. Scale bars: left column (**A**,**C**,**E**,**G**,**I**,**K**,**M**), 400 μm, (magnification 40×); right column (**B**,**D**,**F**,**H**,**J**,**L**,**N**), 10 μm (magnification 400×). * *p* < 0.05 vs. sham, # *p* < 0.05 vs. the corresponding solvent-treated control group.

We further investigated whether the inhibition of Fyn *S*-nitrosylation afforded protective effects against ischemic injury by examining pyramidal neuron survival in the CA1 region with cresyl violet staining. Normal rat neurons were round and pale with intact nuclei (Figure 3A,B), while neurons in rats that underwent 15 min of ischemia were notably smaller and had condensed nuclei five days after reperfusion (Figure 3C,D). Pretreatment of rats with 7-NI (Figure 3I,J), MK-801 (Figure 3K,L) or GSNO (Figure 3M,N) markedly attenuated I/R-induced neuronal degeneration and cell death. However, in comparison, pretreatment of rats with vehicle controls (saline or DMSO) had no effect on I/R-induced damage to neurons (Figure 3E–H).

## 3. Discussion

It has been established that Fyn can be modified by post-translational phosphorylation, myristoylation and palmitoylation. In this paper, we demonstrated that Fyn could be *S*-nitrosylated both in vitro and in vivo. Fyn, when overexpressed in HEK293 cells, was *S*-nitrosylated by GSNO (exogenous NO donor) in a dose-dependent manner. In a rat cerebral I/R model, endogenous NO synthesized by nNOS in brain tissue enhanced the *S*-nitrosylation and phosphorylation of Fyn, while GSNO, 7-NI (a nNOS inhibitor) and MK-801 (an NMDAR antagonist) could decrease the elevated levels of these post-translational modifications induced by cerebral I/R. Morphological analyses using cresyl violet staining showed that these compounds had neuroprotective effects against cerebral I/R-induced injury (Figure 3A,B), which was consistent with previous reports by our laboratory [18,19,20,21].

Recent studies have demonstrated that NO regulates diverse biological and pathological functions of many intracellular signaling proteins [22,23,24]. NO can also modulate many protein functions in a cGMP-independent manner, such as by *S*-nitrosylating proteins [25,26]. Previous work by our laboratory suggested that c-Src *S*-nitrosylation on Cys489, Cys498, and Cys500 can promote c-Src activation and NMDA Receptor 2A (NR2A) phosphorylation via the NMDAR–nNOS module during cerebral I/R [7]. Homology modeling revealed that Fyn has a similar structure to Src, including nine cysteine residues, namely Cys3, Cys6, Cys239, Cys240, Cys246, Cys404, Cys487, Cys491, and Cys502. We therefore investigated whether the same modification can occur in Fyn. In this study, we treated HEK293 cells overexpressing Fyn with GSNO and found that Fyn was *S*-nitrosylated as well (Figure 1A–D). We further confirmed these results by treating HEK293 cells with inactivated GSNO, the absence of ascorbate, or the addition of NEM (Figure 1E–H).

Endogenous NO is produced by NOS utilizing l-arginine as the substrate during cerebral I/R, and its level correlates with NOS activity. Three different isoforms of the enzyme NO synthase (endothelial NOS (eNOS), nNOS, and inducible NOS (iNOS)) are responsible for its synthesis. nNOS and iNOS are mainly present in the brain, and nNOS performs approximately 90% of the NOS activity in normal rodents [27]. Many studies have demonstrated that during ischemia/reperfusion, nNOS and NMDAR, via the scaffolding protein PSD-95, form an NMDAR–PSD-95–nNOS signaling complex and that calcium overload, as a major mechanism of ischemic brain injury, is considered a perpetrator of damage. nNOS is activated upon NMDAR-mediated calcium influx and its interaction with PSD-95, leading to NO production [8,9]. Studies in our laboratory have shown that cerebral I/R may promote the formation of the GluR6–PSD-95–nNOS signaling complex and the activation of GluR5–PSD-95–nNOS signaling module, leading to excessive Ca^2+^ influx, nNOS activation, and subsequent NO release [18,28]. It has been demonstrated that nNOS mediated early neuronal injury (cerebral I/R 6 h following reperfusion) [18,29], whereas later stages of this process were dependent on iNOS (which has detectable protein expression and catalytic activity 12 h after cerebral ischemia) [30,31,32]. In addition, nNOS activity is regulated by Ca^2+^ and calmodulin, whereas iNOS activation does not depend on intracellular Ca^2+^ concentrations but is induced by inflammatory mediators [33]. Therefore, in this study, we mainly focused on the regulation of *S*-nitrosylation by nNOS, and to avoid interference from iNOS, we adopted a 6 h time point after reperfusion.

We utilized 7-NI, a nNOS inhibitor, to investigate the effect of endogenous NO on Fyn *S*-nitrosylation. We found that Fyn *S*-nitrosylation was inhibited in vitro (Figure 1I,J) and in vivo (Figure 2C,D), suggesting that nNOS can mediate Fyn *S*-nitrosylation through the production of endogenous NO. Consistent with this observation, we determined that the NMDA receptor (calcium influx channel) antagonist MK-801 inhibited Fyn *S*-nitrosylation (Figure 2E,F), suggesting potential NMDAR involvement in this process. MK-801, acting on the NMDAR as an open channel blocker, can attenuate NO production by nNOS and inhibit the downstream activation of c-Jun N-terminal kinase 3 (JNK3), mixed lineage kinase 3 (MLK3) and p38 mitogen-activated protein kinases (p38) in rat hippocampus during cerebral I/R. Upon MK-801 treatment, the *S*-nitrosylation of JNK3, MLK3 and p38 was inhibited, and neuronal apoptosis was also suppressed [19,20,34]. In this study, our results indicated that MK-801 inhibited the *S*-nitrosylation of Fyn (Figure 2 and Figure 3).

However, in the present study, we found that the *S*-nitrosylation of Fyn was negatively regulated by GSNO during ischemia–reperfusion (Figure 2E,F). This is very interesting. How can we account for the contradictory phenomenon that a NO donor and an endogenous NOS inhibitor had similar inhibitory effects on the *S*-nitrosylation of Fyn? GSNO itself cannot directly enter cells but may transfer its •NO to cysteine or cystine, forming *S*-nitrosocysteine, which can enter cells; NO freed from *S*-nitrosocysteine can then mediate NO signaling [35]. There is evidence that in many conditions GSNO can increase intracellular *S*-nitrosothiol levels and *S*-nitrosylated protein targets [36,37]. NMDAR located on the cell membrane might be preferentially *S*-nitrosylated by GSNO. Its *S*-nitrosylation can reduce excessive Ca^2+^ influx [38] and thus decrease nNOS activity during cerebral ischemia. NMDAR is particularly effective at inducing calcium/calmodulin-dependent nNOS-mediated production of NO, and this effect is calcium-source-specific, probably via the GluR5–PSD-95–nNOS signaling module in neurons [28,39]. This means that GSNO may *S*-nitrosylate the NMDAR, thus indirectly limiting nNOS activity. In addition, recent results demonstrated that the activity of nNOS is regulated via its phosphorylation (Ser1412) and denitrosylation (Cys331) [40]. Under excitotoxic conditions, GSNO can directly inhibit the activity of nNOS by means of *S*-nitrosylating nNOS at Cys331 [40,41] and mediate the mechanism the dephosphorylation of nNOS at Ser1412 in inhibiting aberrant nNOS [41,42]. From this perspective, the effect of GSNO can be considered as the activity of NMDA receptor or nNOS inhibitor. More importantly, large data demonstrate that in vivo experiments the mechanism of endogenous NO (from NOS) *S*-nitrosylating target proteins differs from that of exogenous NO (GSNO/sodium nitroprusside (SNP)) during I/R in the rat brain [7,19,20,21,28,34]. We inferred that exogenous NO donor decrease the elevated level of Fyn *S*-nitrosylation via antagonizing the effect of endogenous NO produced mostly from nNOS, and/or inhibiting endogenous NO synthesized by NOS. However, it remains to be determined by further research.

The catalytic function of Fyn is tightly regulated by phosphorylation [43]. When a tyrosine residue in Fyn at position 420 was dephosphorylated by tyrosine phosphatase, the enzymatic activity of this protein was inhibited [44,45]. In the present study, we observed that, during cerebral I/R, the nNOS inhibitor (7-NI), the NMDAR antagonist (MK-801), and the NO donor (GSNO) may suppress not only the *S*-nitrosylation but also the phosphorylation of Fyn. The relationship between the *S*-nitrosylation and phosphorylation of Fyn has remained unclear. The drugs (GSNO, 7-NI, and MK-801) that can decrease endogenously produced NO markedly inhibited the phosphorylation of Fyn. One possible mechanism is that Fyn *S*-nitrosylation might affect the phosphorylation of Fyn, but this requires further precise elucidation. Because phosphorylation of Fyn regulates its activation, inhibiting Fyn *S*-nitrosylation may suppress subsequent activation. In the present study, morphological analyses validated that the drugs (7-NI, MK-801 and GSNO) that could inhibit Fyn *S*-nitrosylation also conferred neuronal protection after ischemia in vivo (Figure 3).

Protein *S*-nitrosylation, which serves as a switch for protein function in cell signaling pathways, is a dynamic, specifically targeted, and reversible post-translational modification. Given the specificity of *S*-nitrosylation, not every cysteine-containing protein is *S*-nitrosylated, nor does this process occur at every cysteine residue in a particular protein [46,47]. Thus, future studies merit the investigation of which cysteine(s) in Fyn are *S*-nitrosylated.

It is well known that cerebral ischemia increases the release of glutamate, which activates NMDAR and leads to subsequent nNOS activation and continuous NO production [30,48]. NO, in turn, induces the *S*-nitrosylation, phosphorylation, and activation of Fyn. Activated Fyn then reciprocally regulates the phosphorylation and trafficking of NMDARs, acting as a point of convergence regulating this class of glutamate receptors for numerous signaling pathways [16]. Our results suggest that inhibiting *S*-nitrosylation and subsequent activation of Fyn may indirectly suppress excessive NMDAR activation. The inhibition of Fyn *S*-nitrosylation may therefore have significant neuroprotective roles on the brain damage induced by cerebral I/R, providing a potential therapy for stroke.

## 4. Materials and Methods

### 4.1. Antibodies and Reagents

In our experiments, the following primary antibodies were used: mouse monoclonal Fyn antibody (15) (sc-434, western blot (WB): 1:1000; immunoprecipitation (IP): 2 μg per 400 μg protein) and rabbit polyclonal anti-actin (H-300) antibody (sc-10731, WB: 1:1000) were obtained from Santa Cruz Biotechnology, Inc. (Santa Cruz, CA, USA). Anti-phospho-Fyn (Phospho-Tyr530, E11-0430A) (WB: 1:500) was acquired from EnoGene Biotech Co., Ltd. (New York, NY, USA). Rabbit polyclonal anti-SNO-Cys (N5411) (WB: 1:2000), goat anti-mouse IgG-alkaline phosphatase antibody (A3562, WB: 1:20,000), and goat anti-rabbit IgG-alkaline phosphatase antibody (A3687, WB: 1:20,000) were purchased from Sigma–Aldrich (St. Louis, MO, USA).

Wild-type pcDNA3.1-Fyn plasmid was purchased from Shanghai Generay Biotech Co., Ltd. (Shanghai, China), and pME18s–nNOS expression plasmid was a kind gift from Professor Yasuo Watanabe (Department of Pharmacology and Ophthalmology, Nagoya University School of Medicine, Showa-ku, Nagoya, Japan). The human embryonic kidney 293 (HEK293) cell line was acquired from the Chinese Academy of Sciences (Shanghai, China). High-glucose Dulbecco’s modified Eagle medium (DMEM) and B-27 were from Life Technologies (Rockville, MD, USA). After transfection as described below, HEK293 cells were screened for stable expression of Fyn. The compounds 7-NI (#N7778), GSNO (#N4148), MK-801 (#M107), *N*-ethylmaleimide (NEM) (#E1271), glycine (Gly), poly-l-lysine and streptavidin-agarose were obtained from Sigma–Aldrich (Shanghai, China). All other reagents were from Sigma–Aldrich unless otherwise noted.

### 4.2. Cell Culture and Plasmid Transfection Analysis

HEK293 cells were cultured in DMEM containing 10% fetal calf serum at 37 °C in a settled environment with 5% CO_2_. pcDNA3.1^+^ (Invitrogen, Shanghai, China) was the Fyn expression vector. According to the manufacturer’s instructions, wild-type pcDNA3.1–Fyn plasmid was transfected into HEK293 cells (approximately 60% transfection efficiency). After being cultured in standard media for 48 h, the cells were used for experiments. HEK293 cells were treated with various concentrations of GSNO for 2 h, with 500 μM GSNO for different durations, or with 250 μM NEM before 30 min of GSNO stimulation, after which they were harvested. To investigate whether Fyn could be *S*-nitrosylated by endogenous NO, pcDNA3.1–Fyn and pME18s–nNOS plasmids at a 1:1 ratio were transiently co-transfected into HEK293 cells. After 48 h of transfection, the cells were treated with 10 μM A23187, 10 μM 7-NI, or both drugs for 1 h and then were collected. The cultured cells were lysed by sonication and subsequently centrifuged at 2000× *g* for 10 min in the cold room. The supernatants were used for experiments and were stored at −80 °C until single use. Protein concentration was assessed by the method of Lowry et al. [49].

### 4.3. Experimental Animals

Approximately 150 adult male Sprague-Dawley (SD) rats (250–300 g, 6–8 weeks old) used for the experiments were obtained from Shanghai Experimental Animal Center (Shanghai, China). The rats were housed in standard cages in a room with a controlled environment (temperature: 21 ± 2 °C; relative humidity: 60%–70%; light period: 06:00–18:00) and had access to food and water ad libitum. After three days of acclimatization, the animals were used in research. To examine the time courses of Fyn expression and *S*-nitrosylation after I/R, 5 time points (0, 30 min, 3 h, 6 h, and 12 h) after 15 min of ischemia were selected. Each time point included 4 surviving and successful SD rats. In our present study, the rats were also randomized into 4 groups: sham, I/R, solvent (dimethyl sulfoxide (DMSO) or normal saline (NS)), and treatment, to evaluate the specific effects of NO on the expression, *S*-nitrosylation and phosphorylation of Fyn. Each group consists of 4 surviving and successful SD rats.

All protocols of this study were approved by the Animal Ethics Committee of Xuzhou Medical University (Approval ID: SCXK (SU) 2010-0003, 25 October 2010), which follows the institutional guidelines.

### 4.4. Induction of Transient Cerebral Ischemia

The transient brain ischemia model was established in SD rats by four-vessel occlusion, as described previously by Pulsinelli [50]. Briefly, under intraperitoneal anesthesia with chloral hydrate (300–350 mg/kg), bilateral vertebral arteries were electrocauterized, and both common carotid arteries were isolated. After recovery for 24 h under fasting conditions, the common arteries were occluded using aneurysm clips. The selected rats for the present experiments matched the following criteria: completely flat electroencephalogram, loss of corneal reflex, and maintenance of dilated pupils. After 15 min of ischemia, the clips were removed, and the carotid artery blood flow was restored for reperfusion. During the whole procedure, a heating pad and heating lamps were used to maintain the rectal temperature at 37 °C. Sham-operated control rats were subjected to the same procedure except occlusion of the carotid artery. In the study, the animal survival rate is approximately 60%–70%, and the model success rate is 70%.

### 4.5. Administration of Drugs

This study used 7-NI, MK-801 and GSNO. Their dosage and administration were based on previous reports by our laboratory [18]. MK-801 (3 mg/kg) was dissolved in 0.9% NaCl and injected intraperitoneally (i.p) 20 min before ischemia. 7-NI (25 mg/kg, dissolved in 1% DMSO with sesame oil) was given to the rats through i.p. injection 20 min before ischemia. GSNO (100 μg/kg, dissolved in 0.9% NaCl) was administered via intracerebroventricular (10 μL; distance from bregma: 1.5 mm lateral, 0.8 mm posterior; 3.8 mm deep) infusion at a rate of 1 μL/min. The control rats were given equal volumes of the corresponding solvent (0.9% NaCl or 1% DMSO).

### 4.6. Sample Preparation

Sprague-Dawley rats were decapitated under anesthesia after reperfusion, and the hippocampal CA1 regions were isolated and quickly frozen in liquid nitrogen. Tissues were mechanically disrupted in ice-cold homogenization buffer using a Teflon pestle. The buffer contains 100 mM KCl, 0.5 mM MgCl_2_, 50 mM NaF, 50 mM 3-(*N*-morpholino) propanesulfonic acid (MOPS), 1 mM ethylenediamine tetraacetic acid (EDTA), 1 mM ethylene glycol-bis(2-aminoethylether)-*N*,*N*,*N*′,*N*′-tetraacetic acid (EGTA), 320 mM sucrose, 0.2 mM dithiothreitol (DTT), 1 mM Na_3_VO_4_, 1 mM benzamidine, 20 mM sodium pyrophosphate, 20 mM β-phosphoglycerol, 1 mM *p*-nitrophenyl phosphate, and enzyme inhibitors: 5 μg/mL leupeptin, 1 mM phenylmethylsulfonyl fluoride (PMSF), 5 μg/mL pepstatin A, and 5 μg/mL aprotinin. The homogenates were centrifuged at 800× *g* for 10 min at 4 °C, and the resulting supernatants were collected. Protein content of supernatants was measured by the methods of Lowry et al. [49], and supernatants were normalized for total protein content and stored at −80 °C.

### 4.7. S-Nitrosylation Assay

Determination of Fyn *S*-nitrosylation was carried out by immunoprecipitation (IP) with anti-Fyn antibody, followed by immunoblotting (IB) with the antibody against SNO-Cys, separately.

### 4.8. Immunoblotting Assay

Equal amounts of protein were separated by sodiumdodecyl sulfate sodium dodecyl sulfate-polyacrylamide gel polyacrylamide gel electrophoresis (SDS-PAGE) and electrotransferred to nitrocellulose membranes (Amersham Biosciences, Buckinghamshire, UK). After blocking for 3 h in Tris–buffered saline with 0.1% Tween-20 (TBST) buffer containing 3% bovine serum albumin, the membranes were incubated with primary antibodies overnight at 4 °C. Membranes were then washed and incubated with alkaline phosphatase-conjugated secondary antibody for 2 h at room temperature and developed using the nitro blue tetrazolium/5-bromo-4-chloro-3-indolyl phosphate (NBT/BCIP) assay kit (Promega, Madison, WI, USA). After immunoblotting, the membranes were scanned, and the band densities were analyzed with LabWorks image analysis software (UVP Inc., Upland, CA, USA).

### 4.9. Histological Analysis

After 5 days of I/R, rats were perfusion fixed with 4% paraformaldehyde in 0.1 M sodium phosphate buffer (PBS pH 7.4) under deep anesthesia (chloral hydrate 350 mg/kg, i.p.). Brains were removed quickly. After post-fixation for 24 h at 4 °C, the brains were embedded in paraffin, and then 5-μm-thick successive coronal sections were prepared using a microtome. After being deparaffinized with xylene, the brain sections were rehydrated with ethanol at graded concentrations of 100%–70% (*v*/*v*), followed by washing with water. For histological examination, alternating sections were stained with cresyl violet (0.1%, *w*/*v*). The morphologic changes were observed under a light microscope. The number of surviving hippocampal CA1 neurons per 1 mm of the intermediate sections was counted as the neuronal density.

### 4.10. Statistical Analysis

Data obtained from 4 independent experiments are presented as the mean ± standard deviation (S.D.) and considered as statistically significant at *p* < 0.05 by ANOVA, followed by Duncan’s new multiple range method or the Newman–Keuls test.

## Abbreviations

DMSO	dimethyl sulfoxide
DTT	dithiothreitol
EDTA	ethylenediamine tetraacetic acid
EGTA	ethylene glycol-bis(2-aminoethylether)-*N*,*N*,*N*′,*N*′-tetraacetic acid
JNK	c-Jun N-terminal kinase
NMDAR	*N*-methyl-d-aspartate receptor
nNOS	neuronal nitric oxide synthase
GSNO	*S*-nitrosoglutathione
TBST	tris-buffered saline with 0.1% Tween 20
7-NI	7-nitroindazole
NBT/BCIP	nitro blue tetrazolium/5-bromo-4-chloro-3-indolyl-phosphate
